# A Comparative Study of Ultrasound Examination of Urinary Tract Performed on Spinal Cord Injury Patients with No Urinary Symptoms and Spinal Cord Injury Patients with Symptoms Related to Urinary Tract: Do Findings of Ultrasound Examination Lead to Changes in Clinical Management?

**DOI:** 10.1100/tsw.2006.382

**Published:** 2006-06-30

**Authors:** Subramanian Vaidyanathan, Peter L. Hughes, Bakul M. Soni

**Affiliations:** ^1^ Regional Spinal Injuries Centre, District General Hospital, Southport, UK; ^2^ Department of Radiology, District General Hospital, Southport, PR8 6PN, U.K

**Keywords:** spinal cord injury, kidney, ultrasound

## Abstract

Findings of ultrasound examination of the urinary tract and changes in clinical management, which were instituted on the basis of ultrasound examination, were compared between two groups of spinal cord injury patients. Group 1 had no urinary symptoms when they underwent the scan, whereas group 2 was comprised of patients with symptoms pertaining to the urinary tract. Between 2000 and 2006, ultrasound examination of the urinary tract was performed in 87 spinal cord injury patients who had no urinary symptoms when they underwent the ultrasound scan. No abnormality was found in 63 patients. The ultrasound scan showed some abnormality of the urinary tract in 24 patients (simple cyst in the kidney: 4; reduced size of a kidney: 3; increased echogenicity of left kidney: 1; prominent extrarenal pelvis and mild calyceal dilatation: 1; slightly dilated renal pelvis and calyceal system: 1; pelvic kidney showing mild hydronephrosis: 1; foetal lobulation of kidney: 2; multicystic kidney with no interval change in the appearance since last examination: 1; 2-cm-diameter parapelvic cyst: 1; small renal calyceal calculus: 5; a little cortical scarring bilaterally: 1; focal renal scar: 2; generalised thinning of renal cortex: 3; increase in renal sinus fat: 3; trabeculated bladder: 2; small vesical diverticulum: 1; mild generalised bladder wall thickening: 1; small residual urine in postvoid scan; 2). No specific interventions were performed in these patients on the basis of ultrasound findings. In Group 2, ultrasound examination revealed serious abnormalities such as hydronephrosis, pyonephrosis, vesical calculi, vesical polyp in 20 of 21 patients, and all 20 patients required therapeutic intervention on the basis of ultrasound scan findings.In conclusion, routine ultrasound examination of the urinary tract in spinal cord injury patients who have no urinary symptoms may not be justifiable in terms of cost effectiveness; limited hospital resources should be directed to spinal cord injury patients with urinary symptoms so that ultrasound examination and therapeutic interventions based on ultrasound findings are carried out expeditiously.

## INTRODUCTION

Ozer and Shannon from McGuire VA Medical Center, Richmond, Virginia[1] performed ultrasound scan of kidneys in 52 unselected spinal cord injury patients who returned to the spinal centre for their annual checkups; 36 patients did not have histories of urinary symptoms and renal ultrasound alone discovered no treatable pathology. In contrast, renal ultrasound did show treatable disease in 2 of 16 patients with urinary symptoms. These authors concluded that effectiveness of the ultrasound scan of kidneys in follow-up of spinal cord injury patients would be maintained in the demonstration of treatable disease by the *selective use of renal sonography only in those patients with histories of urinary symptoms.*

The aim of this pilot study is to find out whether ultrasound examination of the urinary tract in spinal cord injury patients who do not have urinary symptoms when the ultrasound scan is performed is justifiable in our clinical setup. If this pilot study shows that the findings of ultrasound scan do not lead to changes in clinical management, then we may omit routine ultrasound examination of the urinary tract in spinal cord injury patients who do not have symptoms related to the urinary tract. We can then direct health care resources, which are not available ad infinitum, to those spinal cord injury patients with urinary symptoms so that ultrasound examination and therapeutic interventions are carried out promptly in these patients.

## PATIENTS AND METHODS

From the records of the Radiology Department, we prepared a list of spinal unit patients who underwent ultrasound examination of the urinary tract during the past 6 years. Spinal cord injury patients who had no urinary symptoms when they underwent ultrasound examination formed the first group. The second group consisted of spinal cord injury patients who had symptoms related to the urinary tract (passing purulent urine, temperature, rigors, passing blood in urine, severe kidney/bladder pain, recurrent urine infections) when they underwent ultrasound examination of the urinary tract. Findings of ultrasound examination were noted in each case. Clinical notes were reviewed and any therapeutic intervention, which was carried out on the basis of the findings of ultrasound examination of the urinary tract, was noted.

### Technique of Ultrasound Examination

A Consultant Radiologist performed ultrasound examination on spinal cord injury patients. The patient was positioned supine on a trolley for ultrasound scan. A 3.5-MHz convex probe (ATL Ultramark 9) was used for the ultrasound examination until 2001. Subsequently, ultrasound scan was performed with a 5.0-MHz convex probe (Keymed Aloka SSD 5500).

## RESULTS

### Group 1 (Patients with No Symptoms When They Underwent the Scan)

Between 2000 and 2006, ultrasound examination of the urinary tract was performed in 87 spinal cord injury patients who had no symptoms related to the urinary tract when they underwent the ultrasound scan. Of the 87 patients, 65 were males; 22 were females. The year of birth of these patients is given in Table 1. The year in which these patients sustained spinal cord injury is provided in Table 2. The numbers of patients with cervical, thoracic, lumbar, and sacral lesions are listed in Table 3.

**TABLE 1**
**Year of Birth of Spinal Cord Injury Patients, Group 1**

| Year of Birth | Number of Patients |
|---|---|
| Between 1920 and 1929 | 2 |
| Between 1930 and 1939 | 13 |
| Between 1940 and 1949 | 33 |
| Between 1950 and 1959 | 20 |
| Between 1960 and 1969 | 8 |
| Between 1970 and 1979 | 7 |
| Between 1980 and 1989 | 3 |
| Between 1990 and 1999 | 1 |

**TABLE 2**
**Year of Injury of Spinal Cord Injury Patients, Group 1**

| Year of Injury | Number of Patients |
|---|---|
| Between 1950 and 1959 | 4 |
| Between 1960 and 1969 | 13 |
| Between 1970 and 1979 | 12 |
| Between 1980 and 1989 | 12 |
| Between 1990 and 1999 | 19 |
| Between 2000 and 2006 | 27 |

**TABLE 3**
**Level of Injury of Spinal Cord Injury Patients, Group 1**

| Level of Injury | Number of Patients |
|---|---|
| Cervical (C) | 39 |
| Thoracic (T) | 33 |
| Lumbar (L) | 13 |
| Sacral (S) | 2 |

Ultrasound examination of the urinary tract revealed no abnormality (no hydronephrosis, renal scarring, calculi, or tumour) in 63 patients. Ultrasound scan showed some abnormality of the urinary tract in 24 patients. Abnormalities, which were detected by ultrasound examination, are listed in Table 4. Some patients had more than one abnormality. No specific surgical or medical intervention was required in any of these 24 patients on the basis of the ultrasound findings.

**TABLE 4**
**Abnormal Findings**

| Abnormal Findings | Number of Patients |
|---|---|
| Simple cyst in the kidney | 4 |
| Reduced size of a kidney | 3 |
| Some increased echogenicity of the left kidney | 1 |
| Prominent extrarenal pelvis and mild calyceal dilatation | 1 |
| Slightly dilated renal pelvis and calyceal system | 1 |
| Right pelvic kidney showing mild hydronephrosis | 1 |
| Foetal lobulation of kidney | 2 |
| Multicystic kidney (no interval change in the appearance since last examination | 1 |
| Small (2-cm-diameter) parapelvic cyst | 1 |
| Small (4-mm) renal calculus in the lower pole | 2 |
| 4-mm calculus in the upper pole of kidney | 1 |
| 5-mm renal calculus in the mid-pole | 2 |
| A little cortical scarring bilaterally | 1 |
| Focal renal scar | 2 |
| Generalised renal cortical thinning | 3 |
| Some increase in renal sinus fat | 3 |
| Trabeculated bladder | 2 |
| Small (1.2 cm) vesical diverticulum | 1 |
| Mild generalised bladder wall thickening | 1 |
| Small residual urine in postvoid scan | 2 |

## Group 2 (Patients with Symptoms Pertaining to the Urinary Tract)

There were 21 spinal cord injury patients who exhibited urinary symptoms (passing purulent urine, temperature, rigors, passing blood in urine, severe kidney/bladder pain, recurrent urine infections) when they underwent ultrasound examination of the urinary tract. Abnormalities such as hydronephrosis, pyonephrosis, vesical calculi, or vesical polyp were detected in 20 of 21 patients and, subsequently, all 20 patients required therapeutic intervention on the basis of ultrasound scan findings.

Findings of ultrasound examination of the urinary tract and changes in clinical management of the urinary tract, which were implemented in these patients on the basis of ultrasound examination, are summarised below.

In five patients with temperature and rigors, ultrasound examination of the urinary tract revealed severe hydronephrosis, and these patients underwent percutaneous nephrostomy. A tetraplegic patient with a history of left periureteral fibrosis and hydronephrosis developed temperature and rigors; ultrasound examination revealed moderate left-sided hydronephrosis with mildly reflective urine in the collecting system, suspicious of infection. Percutaneous renal puncture, collection of urine from renal pelvis for microbiology, and antegrade pyelography were carried out. Antegrade pyelography showed free flow of contrast into the bladder, thus averting a need for nephrostomy. Urine grew *Proteus* sp., sensitive to ciprofloxacin, and this patient was prescribed ciprofloxacin. Subsequently, left ureteric stenting was performed.

In two patients with recurrent urine infection, ultrasound examination revealed marked bilateral hydronephrosis and moderate distension of the urinary bladder. These patients were advised to perform intermittent catheterisation. Subsequent ultrasound examination revealed moderate bilateral hydronephrosis; bladder outline appeared normal. One patient was prescribed oxybutynin whereas the other was started on solifenacin succinate; both patients were given an appointment for videourodynamics. Ultrasound examination in a paraplegic patient with recurrent urine infection revealed mild bilateral hydronephrosis. Cystoscopy showed no tumour or calculi; this patient was advised to perform self-catheterisations.

For a tetraplegic patient who was feeling unwell and had difficulty in voiding, ultrasound examination revealed bilateral moderate hydronephrosis; the renal pelvis was not significantly dilated separate from the calyces and so appearances were presumed to be due to either distal ureteric or bladder outlet obstruction. This patient was advised intermittent catheterisation along with anticholinergic agents following videourodynamics. Ultrasound examination, which was performed 15 months later, showed mild left-sided hydronephrosis; no dilatation on the right side. In a tetraplegic patient with penile sheath drainage and recurrent urine infections, ultrasound examination revealed mild to moderate right hydronephrosis, normal left kidney, and slightly trabeculated bladder outline. The urine in the dilated right pelvicalyceal system was hyporeflective with no definite evidence of a pyonephrosis. This patient was prescribed oxybutynin and terazosin, and advised to have intermittent catheterisations three times a day.

In another tetraplegic patient with recurrent urine infections, ultrasound examination revealed multiple cystic areas within the right kidney. Ultrasonically, it was difficult to say whether these were due to multiple cysts or due to dilated calyces. The ureter was not visualised due to overlying bowel gas, but there was no evidence of any pyonephrosis. This patient was prescribed antibiotics and a follow-up ultrasound examination revealed marked right hydronephrosis with a very thin rim of renal cortex; left kidney ultrasonically appeared normal. MAG-3 renogram showed no noticeable function in the right kidney; there was normal uptake and excretion in the left kidney. Relative function of left kidney: 100%; right kidney: 0%. Therefore, this patient was advised to undergo right nephrectomy.

In a tetraplegic patient with history of passing blood in urine, ultrasound examination revealed no hydronephrosis, but the bladder was very thick walled and there appeared to be a polyp arising from the bladder wall inferiorly. Cystoscopy was performed.

In four patients who had frequent catheter blocks, ultrasound examination revealed bladder calculi in three, and a lot of debris within the bladder was noted in the fourth patient. These patients underwent cystoscopy and removal of calculi and debris.

In a paraplegic patient with recurrent urine infections, ultrasound examination revealed slight cortical thinning in the upper pole of left kidney where a 1-cm-diameter calculus was present. X-ray of the abdomen, taken a week later, showed opaque calculus, projected over the upper pole of left kidney, and opaque calculus projected over the right renal pelvis. This patient was advised 24-h urine biochemistry for calcium, phosphate, oxalate, and citrate. The renal calculi would be treated by shock wave lithotripsy.

In a 24-year-old paraplegic patient with recurrent urine infections, ultrasound examination revealed a small calculus in the mid-pole region of right kidney and further small calculus in the mid-pole region of left kidney. This patient was advised medical therapy for nephrolithiasis (high fluid intake, moderate restriction of animal protein and sodium chloride, potassium intake to be encouraged, and calcium restriction to be avoided while intake of calcium and oxalate to be balanced).

A 62-year-old male with paraplegia and gout developed recurrent urine infections. Ultrasound examination revealed multiple cysts replacing much of the renal parenchyma of both kidneys. There was dilatation of right renal pelvis and a large stag horn type calculus in dilated lower pole calyces of right kidney. Smaller calculi were seen in the lower pole of left kidney, which was also hydronephrotic. This patient is currently undergoing further radiological and biochemical investigations.

A paraplegic patient underwent ultrasound examination of the urinary tract because he was running a temperature. The ultrasound scan showed normal kidneys; no calculi, scarring, or hydronephrosis; normal liver parenchyma; splenomegaly at 14 cm. There was thrombosis of the left common iliac and external iliac veins. This patient was started on anticoagulants and made an uneventful recovery.

In a tetraplegic patient with a past history of bladder stones and pain lower abdomen, ultrasound examination revealed normal kidneys, no residual bladder calculi. This patient was reassured.

## DISCUSSION

### *Is ultrasound examination of the urinary tract cost effective for spinal cord injury patients who have no urinary symptoms?*

Sipski and associates from the South Florida Model Spinal Cord Injury System, University of Miami School of Medicine, Florida[2] carried out a retrospective review of the charts of 174 individuals with spinal cord injury or spinal cord disorder who underwent abdominal ultrasound testing between 1997 and 2000 during their routine annual evaluation. Ultrasound examination revealed kidney abnormalities in 7 of 174 patients. The renal abnormalities detected by ultrasound were: solitary cysts (5.6%), abnormal size (0.6%), and combined pathology – cyst and abnormal size (0.6%). Interestingly, no hydronephrosis was reported. No specific interventions were noted solely on the basis of the ultrasound findings. *These authors concluded that renal abnormalities were infrequent in their patients and performing annual renal sonography to detect treatable pathology in spinal cord injury patients would not be cost effective.*

In this study, ultrasound examination of the urinary tract was performed on 87 spinal cord injury patients who had no urinary symptoms. Some abnormality was detected in 24 patients, but no therapeutic intervention was instituted in any of these patients on the basis of ultrasound findings. In contrast, ultrasound examination of the urinary tract proved to be a very valuable investigation in spinal cord injury patients with urinary symptoms, as 20 of 21 patients required therapeutic interventions on the basis of the findings of ultrasound scan.

### *Should renal sonography be used selectively in patients with urinary symptoms in order to lower the cost for surveillance of spinal cord injury patients?*

Ozer and Shannon[1] recommended selective use of renal sonography in spinal cord injury patients with histories of urinary symptoms since in no instance did sonography alone identify presence of a disease in the absence of histories of genitourinary symptoms.

We found that ultrasound examination of the urinary tract in spinal cord injury patients who did not have urinary symptoms when they underwent ultrasound scan revealed no abnormality in 63 of 87 patients. Ultrasound examination revealed some abnormalities in 24 patients, but detection of abnormality did not warrant changes in clinical management in these patients. In contrast, ultrasound examination of the urinary tract in patients *with* symptoms pertaining to the urinary tract such as temperature, rigors, passing blood in urine, or recurrent urine infection, revealed pathologies in 20 of 21 patients, who required therapeutic interventions based on the findings of ultrasound examination. *Our observations would support the recommendation made by Ozer and Shannon that greater selectivity in the use of sonography would ensure that cost effectiveness of the annual follow-up of spinal cord injury patients was maintained.*

### *Is compliance with annual urologic evaluations associated with preservation of renal function in spinal cord injury patients?*

Waites and associates from the University of Alabama at Birmingham[3] recommended that spinal injury units should carefully evaluate protocols regarding follow-up urologic examinations in persons with spinal cord injury in order to provide the most cost-effective programme while maintaining optimum patient care. These authors compared 59 subjects with spinal cord injury who had missed two or more consecutive annual examinations and 101 controls with spinal cord injury who were compliant with routine annual examinations for the previous three consecutive years. Effective renal plasma flow (ERPF) was measured in both groups of patients. There was no significant difference in age-adjusted mean ERPF between subjects and controls with spinal cord injury. *Renal function was adequately preserved in the majority of persons with spinal cord injury irrespective of compliance with follow-up.* This study raised a fundamental question as to whether annual urologic evaluations were indeed necessary in spinal cord injury patients after the first few years postinjury and whether it would be acceptable to lengthen the periods between examinations. Our initial study, reported herein, shows ultrasound examination of the urinary tract in spinal cord injury patients who do not have urinary symptoms does not lead to changes in clinical management and, therefore, may not be justifiable in terms of cost effectiveness. As the next step, we propose to compare spinal cord injury patients who underwent annual urologic evaluations and those who did not to find out whether renal function is preserved equally in both groups, or if treatable pathologies exist in patients who missed annual urologic evaluations.

### *Should spinal cord physicians develop a system of care that is beneficial to the patient and cost effective to society by adopting plans for surveillance that are based on clinical status of individual patients and patients' ability to self-monitor their health?*

Kirkman-Liff[4] advocated the concept of rationing based on clinical and epidemiological information. This policy involves targeting screening or treatment to those for whom the screening would most benefit and not providing it to those for whom the screening would least benefit. The National Health Service of United Kingdom is facing a total end of year (March 2006) deficit of £800m ($1.4bn), as predicted by health economists[5]; therefore, it may be a wise decision to use available resources in the most cost-effective manner. At present, in our spinal unit, the waiting time for ultrasound examination of the urinary tract of a spinal cord injury patient who is not admitted to spinal unit is 20 weeks. The resources required for performing ultrasound examination of the urinary tract on spinal cord injury patients are significant (ultrasound scan facility, radiologists' time, hospital ambulance facility, which is required to bring a tetraplegic patient from the residential home to hospital, spinal unit staff time required for hoisting a tetraplegic patient on to a trolley, hospital porters' time in transporting spinal cord injury patients from spinal unit to radiology department and back to spinal unit). By selective use of ultrasound examination, we should be able to direct the limited resources to those spinal cord injury patients who have developed urinary symptoms, e.g., recurrent urine infections, passing blood in urine, severe kidney/bladder pain, temperature, rigors, passing purulent urine. With this approach, patients with symptoms pertaining to the urinary tract will be able to undergo ultrasound examination expeditiously. Prompt investigation will, in turn, facilitate early diagnosis and treatment of urinary tract ailments before complications occur, as a long waiting period for investigations often results in preventable complications such as worsening of hydronephrosis, development of pyonephrosis, increase in the size and number of bladder, or renal calculi.

McMahon and associates[6] recommend the use of multivariate risk assessment instead of the univariate solution. The current "one size fits all" strategy can result in significant overtreatment of low-risk patients or directing too little attention and resources to high-risk patients, or both. For example, 80% of the risk of serious diabetic complications is clustered in 15–20% of patients. Similar findings are available for spinal cord injury patients also. Middleton and associates[7] analysed the frequency, cause, and duration of rehospitalisations in individuals with spinal cord injury living in the community, from the Royal North Shore Hospital (RNSH) Spinal Cord Injuries Database in Australia. Over 50% of readmissions (33 out of 64) for pressure sores occurred in only nine individuals aged 30 years or under. Surprisingly, a small subgroup of young males developed late complication of pressure sores and consumed disproportionately large resources. Review of data for 8668 persons with spinal cord injury from 16 Model Spinal Cord Injury Systems centres entered in the National Spinal Cord Injury Statistical Centre database, USA between 1995 and 2002, revealed that diseases of the respiratory system tended to be more likely in patients with tetraplegia (C1-8 American Spinal Injury Association [ASIA] grades A, B, C); whereas patients with paraplegia (T1-S5 ASIA grades A, B, C) were more likely to be rehospitalised for pressure ulcers[8].

We observed that ultrasound examination of the urinary tract in spinal cord injury patients who did not have urinary symptoms when they underwent ultrasound scan did not lead to changes in clinical management of these patients. In contrast, 20 of 21 patients with urinary symptoms required therapeutic intervention on the basis of the findings of ultrasound examination of the urinary tract. These observations support the concept advocated by McMahon and associates[6] that we should wisely utilise the resources entrusted to the medical profession by the society and target our efforts to spinal cord injury patients who are likely to benefit from screening and preventative measures.

As pointed out by McMahon and associates[6], if a health system is responsible for caring for 1000 patients with diabetes, more good will be accomplished if 50 higher-risk patients receive close eye care follow-up than if 700 to 800 low-risk patients meet current “one size fits all” retinal screening quality standards. Most health care systems may be spending greater resources to ensure that all patients with diabetes have annual eye examinations and urine protein screening than on intensive follow-up of high-risk patients with diabetes and known retinopathy. In spinal cord injury medicine also, identifying rates, causes, and patterns of morbidity is important for future resource allocation and targeting preventative measures. For instance, the late complication of pressure sores in a small subgroup of young males, consuming disproportionately large resources, warrants further research to better understand the complex psychosocial and environmental factors involved and to develop effective countermeasures[7].

As stated by Sipski and associates[2], implementing a lifetime care plan for spinal cord injury patients requires development of a system of care that is beneficial to the patient and cost effective to society. It is prudent that spinal cord physicians are flexible with the follow-up protocols. Plans for surveillance should be based on the clinical needs of individual patient and patients’ ability to self-monitor their general health. Rigid protocols or routine policy should not dictate what tests are done, and certainly, should not be a substitute for sound clinical judgement. Individualised follow-up protocols will promote increased responsibility and control for both spinal cord injury patient and spinal cord physician.

## Study Limitations

Conclusions of this study are applicable only to *asymptomatic* spinal cord injury patients who come for routine annual follow-up. Health professionals should remember that patients with spinal cord injury may develop hydronephrosis silently[9]. For example, after an apparently successful trial of micturition, some patients may retain progressively increasing volumes of urine in the bladder over a period. The symptoms of unsatisfactory bladder emptying are nonspecific and include (1) urinary infection, (2) sweating, and (3) increased spasms. Therefore, spinal cord injured patients who have completed a successful trial of micturition must have a meticulous follow-up. Urine microbiology, urodynamics, and ultrasound scan of the urinary tract must be performed at regular intervals after a successful trial of micturition[10]. Similarly, patients with spinal cord injury who have developed hydronephrosis or pyonephrosis may present with bizarre and nonspecific symptoms; such symptoms include feeling unwell, abdominal discomfort, increased spasms, and autonomic dysreflexia. Physicians should be aware of the serious import of these symptoms in spinal cord injured patients[11].

Further, this study does not address the issue of laboratory investigations that should be performed during follow-up of spinal cord injury patients. It is possible that estimation of urinary alpha-1-microglobulin excretion may prove useful to assess the grade of vesicoureteral reflux, detrusor pressure, and compliance[12]. In a study from the U.S. Department of Veterans Affairs, Choi and associates evaluated laboratory components of a comprehensive periodic, health evaluation programme for patients with spinal cord injury and disorders[13]. Although abnormality rates for many routine laboratory tests were high (up to 31.5%), diagnostic and therapeutic yields were low (<1.5%), with the exception of glucose (therapeutic yield, 3.4%) and lipid tests (up to a 4.1% diagnostic and 15.2% therapeutic yield). Sepahpanah and associates showed that the creatinine clearance test had little value as a screening measure for renal disease in spinal cord injury patients because of its variability in serial testing[14]. These authors suggested that a new method of determining creatinine clearance in spinal cord injury patients that involved the measurement of serum cystatin might be a more appropriate and sensitive determinant of renal function in this population. Debate thus continues as to the most appropriate laboratory tests that should be performed for evaluation of the urinary tract during follow-up of spinal cord injury patients.

Lastly, our study focused on whether results of ultrasound examination of the urinary tract in *asymptomatic* spinal cord injury patients led to changes in clinical management. We did not investigate the relative merits of ultrasound examination vs. isotope scan in evaluation of urinary tract during follow-up of spinal cord injury patients. Similarly, identification of patients who were at risk for developing urinary tract complications on the basis of age, gender, degree of spinal cord damage (ASIA scale A, B, C or D), level of spinal cord injury, or number of years after sustaining spinal cord injury, fell outside the remit of this study.

## CONCLUSION

Results of ultrasound examination of the urinary tract, performed in 87 spinal cord injury patients who did not have symptoms related to the urinary tract at the time of undergoing the scan showed no abnormal findings in 63 cases. Ultrasound examination revealed some abnormal findings in 24 patients, but detection of abnormal findings did not lead to changes in clinical management of these patients. In contrast, 20 of 21 patients with urinary symptoms who underwent ultrasound examination of urinary tract required therapeutic intervention on the basis of the findings of ultrasound scan. Therefore, routine ultrasound examination of the urinary tract in spinal cord injury patients who have no urinary symptoms may not be justifiable in terms of cost effectiveness; limited hospital resources should be directed to spinal cord injury patients with urinary symptoms so that ultrasound examination and therapeutic interventions based on ultrasound findings are carried out expeditiously.

## ACKNOWLEDGEMENT

The authors are grateful to Dr. Z. Kirkali, Editorial Board Member of *TSW Urology* for valuable advice.

## References

1. Ozer, M.N. and Shannon, S.R. (1991) Renal sonography in asymptomatic persons with spinal cord injury: aA cost-effectiveness analysis. *Arch. Phys. Med. Rehabil.* **72,** 35–37.
2. Sipski, M.L., Estores, I.M., Alexander, C.J., Guo, X., and Chandralapaty, S.K. (2004) Lack of justification for routine abdominal ultrasonography in patients with chronic spinal cord injury. *J. Rehabil. Res. Dev.* **41,** 101–108.
3. Waites, K.B., Canupp, K.C., DeVivo, M.J., Lloyd, L.K., and Dubovsky, E.V. (1995) Compliance with annual urologic evaluations and preservation of renal function in persons with spinal cord injury *J. Spinal Cord Med.* **18,** 251–254.
4. Kirkman-Liff, B. (2005) Rationing healthcare based on clinical and epidemiological information. *Am. J. Manag. Care* **11**, 658.
5. Day, M. (2006) NHS faces job cuts as financial crisis deepens. *BMJ* **332**, 743.
6. McMahon, L.F., Jr., Hayward, R., Saint, S., Chernew, M.E., and Fendrick, A.M. (2005) Univariate solutions in a multivariate world: can we afford to practice as in the "good old days"? *Am. J. Manag. Care* **11**, 473–474.
7. Middleton, J.W., Lim, K., Taylor, L., Soden, R., and Rutkowski, S. (2004) Patterns of morbidity and rehospitalisation following spinal cord injury. *Spinal Cord* **42**, 359–367.
8. Cardenas, D.D., Hoffman, J.M., Kirshblum, S., and McKinley, W. (2004) Etiology and incidence of rehospitalization after traumatic spinal cord injury: a multicenter analysis. *Arch. Phys. Med. Rehabil.* **85**, 1757–1763.
9. Rosen, J.S., Nanninga, J.B., and O'Conor, V.J. (1976) Silent hydronephrosis, a hazard revisited. *Paraplegia* **14,** 124–129.
10. Vaidyanathan, S., Soni, B.M., Sett, P., Singh, G., Oo, T., Hughes, P.L., and Mansour, P. (2003) Flawed trial of micturition in cervical spinal cord injury patients: guidelines for trial of voiding in men with tetraplegia. *Spinal Cord* **41,** 667–672.
11. Vaidyanathan, S., Singh, G., Soni, B.M., Hughes, P.L., Watt, J.W., Dundas, S., Sett, P., and Parsons, K.F. (2000) Silent hydronephrosis/pyonephrosis due to upper urinary tract calculi in spinal cord injury patients. *Spinal Cord* **38**,

661–668.

12. Everaert, K., Van Laecke, E., Dekuyper, P., Hoebeke, P., Delanghe, J., Oosterlinck, W., and Vande Walle, J. (2001) Impact of urinary tract infection and detrusor pressure on renal tubular function in patients with vesicoureteral reflux. *Eur. Urol.* **39**, 337–342.
13. Choi, H., Binder, D.S., Oropilla, M.L., Bernotus, E.E., Konya, D., Nee, M.A., Tammaro, E.A., and Sabharwal, S. (2006) Evaluation of selected laboratory components of a comprehensive periodic health evaluation for veterans with spinal cord injury and disorders. *Arch. Phys. Med. Rehabil.* **87**, 603–610.
14. Sepahpanah, F., Burns, S.P., McKnight, B., and Yang, C.C. (2006) Role of creatinine clearance as a screening test in persons with spinal cord injury. *Arch. Phys. Med. Rehabil.* **87**, 524–528.
